# 2-(4-Fluoro­phen­yl)-3-methyl-1*H*-indole

**DOI:** 10.1107/S1600536811021325

**Published:** 2011-06-11

**Authors:** David B. Cordes, Guoxiong Hua, Alexandra M. Z. Slawin, J. Derek Woollins

**Affiliations:** a School of Chemistry, University of St Andrews, Fife KY16 9ST, Scotland

## Abstract

The indole N—H hydrogen in the title compound, C_15_H_12_FN, does not display classical hydrogen bonding. Rather it forms an interaction with the π system of an adjacent indole, resulting in weakly inter­acting chains along the [001] direction.

## Related literature

The title compound has previously been prepared by Buu-Hoi & Jacquignon (1949 ▶) and by Kraus & Guo (2009 ▶). For the synthesis of the starting material, 4-fluoro-*N*-(2-oxo-2-phenyl­eth­yl)benzamide, see: Moriya *et al.* (1986 ▶). There are no structures of closely related compounds in the literature. For some similar compounds, see: Schmelter *et al.* (1973 ▶); Konno *et al.* (2004 ▶); Kumar & Liu (2006 ▶).
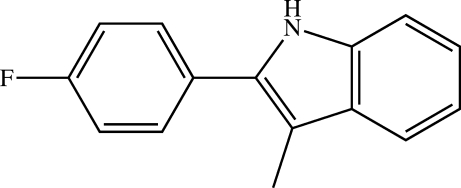


## Experimental

### 

#### Crystal data

C_15_H_12_FN
*M* *_r_* = 225.26
Monoclinic, 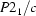

*a* = 7.790 (3) Å
*b* = 17.125 (6) Å
*c* = 8.811 (4) Å
β = 110.274 (9)°
*V* = 1102.7 (8) Å^3^
*Z* = 4
Mo *K*α radiation
μ = 0.09 mm^−1^
*T* = 93 K
0.25 × 0.20 × 0.15 mm

#### Data collection

Rigaku Mercury CCD diffractometer
Absorption correction: multi-scan (*CrystalClear*; Rigaku, 2010 ▶) *T*_min_ = 0.706, *T*_max_ = 1.000
7380 measured reflections
2361 independent reflections
1703 reflections with *I* > 2σ(*I*)
*R*_int_ = 0.049

#### Refinement

*R*[*F* ^2^ > 2σ(*F* ^2^)] = 0.065
*wR*(*F* ^2^) = 0.195
*S* = 1.07
2361 reflections
160 parameters
1 restraint
H atoms treated by a mixture of independent and constrained refinement
Δρ_max_ = 0.31 e Å^−3^
Δρ_min_ = −0.21 e Å^−3^

### 

Data collection: *CrystalClear* (Rigaku, 2010 ▶); cell refinement: *CrystalClear*; data reduction: *CrystalClear*; program(s) used to solve structure: *SHELXTL* (Sheldrick, 2008 ▶); program(s) used to refine structure: *SHELXTL*; molecular graphics: *SHELXTL*; software used to prepare material for publication: *SHELXTL*.

## Supplementary Material

Crystal structure: contains datablock(s) global, I. DOI: 10.1107/S1600536811021325/si2359sup1.cif

Structure factors: contains datablock(s) I. DOI: 10.1107/S1600536811021325/si2359Isup2.hkl

Supplementary material file. DOI: 10.1107/S1600536811021325/si2359Isup3.cml

Additional supplementary materials:  crystallographic information; 3D view; checkCIF report

## Figures and Tables

**Table 1:** Hydrogen-bond geometry (Å, °) *Cg* is the centroid of the C3–C8 ring.

*D*—H⋯*A*	*D*—H	H⋯*A*	*D*⋯*A*	*D*—H⋯*A*
N1—H1⋯*Cg*^i^	0.94 (2)	2.99 (2)	3.791 (2)	143 (2)

Symmetry code: (i) 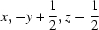.

**Fig. 1.**
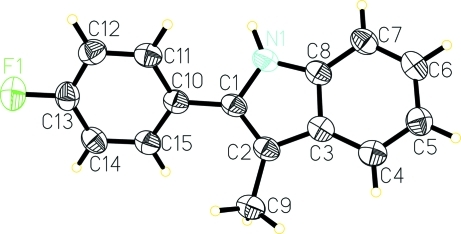
The molecular structure of the title compound with displacement ellipsoids drawn at the 50% probability level.

## supplementary crystallographic information

### Comment

The previously known title compound (Buu-Hoi & Jacquignon, 1949 and Kraus & Guo, 2009) has been synthesized by a new route from Woollins' reagent and 4-fluoro-*N*-(2-oxo-2-phenylethyl)benzamide. The structure of the compound is not closely related to any known structures; however, among those structures showing similarities (Schmelter *et al.*, 1973, Konno *et al.*, 2004, and Kumar & Liu, 2006) the indole N—H shows a tendency toward interactions with π systems, rather than conventional hydrogen bonding, similar to that observed in the title compound.

### Experimental

A suspension of 4-fluoro-*N*-(2-oxo-2-phenylethyl)benzamide (0.26 g, 1.0 mmol, Moriya *et al.*1986) and Woollins' reagent (0.54 g, 1.0 mmol) in 20 ml of dry toluene was refluxed for 7 h. Following cooling to room temperature and removal of solvent *in vacuuo* the residue was purified by silica gel column chromatography (dichloromethane eluent) to give the title compound as a bright grey solid (0.140 g, 62%). Crystals suitable for X-ray structure determination were obtained from the diffusion of hexane into a dichloromethane solution of the title compound. *M*.p. 140 - 141 °C. Selected IR (KBr, cm^-1^): 3423(s, NH), 1563(*m*), 1458(*m*), 1332(*m*), 1304(*m*), 1218(*s*), 1154(*m*), 838(*s*), 746(*s*), 468(*m*). ^1^H NMR (CD_2_Cl_2_, δ), 8.07 (s, 1H, NH), 7.56 (m, 2H, ArH), 7.35 (d, *J* = 7.7 Hz, 1H, ArH), 7.22–7.09 (m, 5H, ArH), 2.42 (s, 3H, CH_3_) p.p.m.. ^13^C NMR (CD_2_Cl_2_, δ), 164.0 (C—F), 135.9, 133.1, 129.6, 129.4, 122.3, 119.5, 118.9, 115.9, 115.6, 110.7, 108.5, 9.4 (CH_3_) p.p.m.. MS (CI^+^, m/*z*), 226 [*M*+H]^+^. MS (EI^+^, m/*z*), 225 [*M*]^+^. Accurate mass measurement [EI^+^, m/*z*]: 224.0870 [M—H]^+^, calculated mass for C_15_H_11_NF: 224.0870.

### Refinement

The NH hydrogen atom was located from the difference fourier map and refined isotropically subject to a distance restraint (N—H = 0.98 Å). Carbon-bound H atoms were included in calculated positions (C—H distances are 0.98 Å for methyl H atoms and 0.95 Å for phenyl H atoms) and refined as riding atoms with *U*_iso_(H) = 1.2 *U*_eq_(parent atom, phenyl H atoms) or *U*_iso_(H) = 1.5 *U*_eq_ (parent atom, methyl H atoms).

### Crystal data

**Table:**

C_15_H_12_FN	*F*(000) = 472
*M**_r_* = 225.26	*D*_x_ = 1.357 Mg m^−^^3^
Monoclinic, *P*2_1_/*c*	Mo *K*α radiation, λ = 0.71073 Å
Hall symbol: -P 2ybc	Cell parameters from 3217 reflections
*a* = 7.790 (3) Å	θ = 2.4–28.3°
*b* = 17.125 (6) Å	µ = 0.09 mm^−^^1^
*c* = 8.811 (4) Å	*T* = 93 K
β = 110.274 (9)°	Platelet, colorless
*V* = 1102.7 (8) Å^3^	0.25 × 0.20 × 0.15 mm
*Z* = 4	

### Data collection

**Table:**

Rigaku Mercury CCD diffractometer	2361 independent reflections
Radiation source: rotating anode	1703 reflections with *I* > 2σ(*I*)
confocal	*R*_int_ = 0.049
Detector resolution: 14.7059 pixels mm^-1^	θ_max_ = 28.7°, θ_min_ = 2.4°
ω and φ scans	*h* = −9→9
Absorption correction: multi-scan (*CrystalClear*; Rigaku, 2010)	*k* = −16→23
*T*_min_ = 0.706, *T*_max_ = 1.000	*l* = −11→10
7380 measured reflections	

### Refinement

**Table:**

Refinement on *F*^2^	Secondary atom site location: difference Fourier map
Least-squares matrix: full	Hydrogen site location: inferred from neighbouring sites
*R*[*F*^2^ > 2σ(*F*^2^)] = 0.065	H atoms treated by a mixture of independent and constrained refinement
*wR*(*F*^2^) = 0.195	*w* = 1/[σ^2^(*F*_o_^2^) + (0.1147*P*)^2^] where *P* = (*F*_o_^2^ + 2*F*_c_^2^)/3
*S* = 1.07	(Δ/σ)_max_ < 0.001
2361 reflections	Δρ_max_ = 0.31 e Å^−^^3^
160 parameters	Δρ_min_ = −0.21 e Å^−^^3^
1 restraint	Extinction correction: *SHELXTL* (Sheldrick, 2008), Fc^*^=kFc[1+0.001xFc^2^λ^3^/sin(2θ)]^-1/4^
Primary atom site location: structure-invariant direct methods	Extinction coefficient: 0.052 (13)

### Special details

**Table:**

Geometry. All e.s.d.'s (except the e.s.d. in the dihedral angle between two l.s. planes) are estimated using the full covariance matrix. The cell e.s.d.'s are taken into account individually in the estimation of e.s.d.'s in distances, angles and torsion angles; correlations between e.s.d.'s in cell parameters are only used when they are defined by crystal symmetry. An approximate (isotropic) treatment of cell e.s.d.'s is used for estimating e.s.d.'s involving l.s. planes.
Refinement. Refinement of *F*^2^ against ALL reflections. The weighted *R*-factor *wR* and goodness of fit *S* are based on *F*^2^, conventional *R*-factors *R* are based on *F*, with *F* set to zero for negative *F*^2^. The threshold expression of *F*^2^ > σ(*F*^2^) is used only for calculating *R*-factors(gt) *etc*. and is not relevant to the choice of reflections for refinement. *R*-factors based on *F*^2^ are statistically about twice as large as those based on *F*, and *R*- factors based on ALL data will be even larger. A distance restraint was applied to the N—H bond.

### Fractional atomic coordinates and isotropic or equivalent isotropic displacement parameters (Å^2^)

**Table:**

	*x*	*y*	*z*	*U*_iso_*/*U*_eq_	
F1	0.44463 (18)	0.36586 (7)	0.14270 (16)	0.0629 (5)	
N1	−0.1358 (2)	0.33607 (9)	0.5104 (2)	0.0416 (5)	
H1	−0.190 (3)	0.3064 (13)	0.416 (2)	0.070 (7)*	
C1	0.0281 (2)	0.37720 (10)	0.5467 (2)	0.0373 (5)	
C2	0.0708 (2)	0.41011 (10)	0.6983 (2)	0.0392 (5)	
C3	−0.0707 (2)	0.38743 (10)	0.7584 (2)	0.0386 (5)	
C4	−0.1007 (3)	0.40028 (10)	0.9045 (2)	0.0427 (5)	
H4	−0.0164	0.4305	0.9878	0.051*	
C5	−0.2529 (3)	0.36883 (11)	0.9262 (2)	0.0456 (5)	
H5	−0.2725	0.3767	1.0256	0.055*	
C6	−0.3796 (3)	0.32527 (11)	0.8028 (2)	0.0470 (5)	
H6	−0.4854	0.3053	0.8195	0.056*	
C7	−0.3545 (2)	0.31077 (11)	0.6581 (2)	0.0445 (5)	
H7	−0.4404	0.2809	0.5754	0.053*	
C8	−0.1987 (2)	0.34150 (10)	0.6377 (2)	0.0392 (5)	
C9	0.2314 (3)	0.46041 (11)	0.7870 (2)	0.0466 (5)	
H9A	0.3395	0.4421	0.7648	0.070*	
H9B	0.2545	0.4574	0.9034	0.070*	
H9C	0.2052	0.5146	0.7507	0.070*	
C10	0.1309 (2)	0.37612 (10)	0.4355 (2)	0.0379 (5)	
C11	0.1282 (2)	0.30989 (11)	0.3413 (2)	0.0420 (5)	
H11	0.0543	0.2665	0.3462	0.050*	
C12	0.2309 (2)	0.30642 (11)	0.2413 (2)	0.0456 (5)	
H12	0.2274	0.2616	0.1766	0.055*	
C13	0.3388 (3)	0.36986 (12)	0.2380 (2)	0.0459 (5)	
C14	0.3432 (3)	0.43669 (11)	0.3255 (2)	0.0446 (5)	
H14	0.4166	0.4799	0.3188	0.054*	
C15	0.2382 (2)	0.43944 (10)	0.4235 (2)	0.0415 (5)	
H15	0.2390	0.4854	0.4840	0.050*	

### Atomic displacement parameters (Å^2^)

**Table:**

	*U*^11^	*U*^22^	*U*^33^	*U*^12^	*U*^13^	*U*^23^
F1	0.0641 (9)	0.0696 (9)	0.0655 (9)	0.0056 (7)	0.0357 (7)	0.0017 (7)
N1	0.0344 (9)	0.0434 (9)	0.0440 (10)	−0.0072 (6)	0.0099 (7)	−0.0019 (8)
C1	0.0319 (9)	0.0299 (8)	0.0474 (11)	−0.0007 (7)	0.0101 (8)	0.0018 (8)
C2	0.0325 (9)	0.0340 (10)	0.0482 (11)	0.0003 (7)	0.0104 (8)	0.0014 (8)
C3	0.0349 (9)	0.0319 (9)	0.0461 (11)	0.0048 (7)	0.0104 (8)	0.0052 (8)
C4	0.0434 (11)	0.0340 (10)	0.0484 (12)	0.0027 (8)	0.0128 (9)	0.0020 (9)
C5	0.0476 (12)	0.0420 (11)	0.0487 (12)	0.0023 (8)	0.0183 (10)	0.0052 (9)
C6	0.0396 (11)	0.0448 (11)	0.0579 (13)	0.0000 (8)	0.0187 (9)	0.0100 (10)
C7	0.0363 (10)	0.0451 (11)	0.0479 (12)	−0.0021 (8)	0.0093 (9)	0.0062 (9)
C8	0.0322 (9)	0.0367 (10)	0.0453 (11)	0.0011 (7)	0.0091 (8)	0.0038 (9)
C9	0.0388 (10)	0.0448 (11)	0.0523 (12)	−0.0058 (8)	0.0107 (9)	−0.0037 (10)
C10	0.0315 (9)	0.0369 (10)	0.0412 (11)	0.0024 (7)	0.0071 (8)	0.0024 (8)
C11	0.0378 (10)	0.0382 (10)	0.0450 (11)	−0.0014 (7)	0.0080 (8)	0.0001 (9)
C12	0.0424 (11)	0.0450 (12)	0.0444 (12)	0.0038 (8)	0.0085 (9)	−0.0039 (9)
C13	0.0397 (11)	0.0548 (12)	0.0440 (11)	0.0073 (9)	0.0156 (9)	0.0043 (10)
C14	0.0371 (10)	0.0420 (11)	0.0533 (12)	0.0007 (8)	0.0139 (9)	0.0079 (9)
C15	0.0353 (10)	0.0356 (10)	0.0504 (11)	0.0021 (7)	0.0106 (8)	0.0006 (9)

### Geometric parameters (Å, °)

**Table:**

F1—C13	1.367 (2)	C7—C8	1.391 (3)
N1—C8	1.374 (2)	C7—H7	0.9500
N1—C1	1.395 (2)	C9—H9A	0.9800
N1—H1	0.944 (16)	C9—H9B	0.9800
C1—C2	1.380 (3)	C9—H9C	0.9800
C1—C10	1.465 (3)	C10—C15	1.395 (3)
C2—C3	1.432 (2)	C10—C11	1.402 (3)
C2—C9	1.497 (3)	C11—C12	1.382 (3)
C3—C4	1.403 (3)	C11—H11	0.9500
C3—C8	1.417 (3)	C12—C13	1.380 (3)
C4—C5	1.375 (3)	C12—H12	0.9500
C4—H4	0.9500	C13—C14	1.374 (3)
C5—C6	1.403 (3)	C14—C15	1.381 (3)
C5—H5	0.9500	C14—H14	0.9500
C6—C7	1.378 (3)	C15—H15	0.9500
C6—H6	0.9500		
			
C8—N1—C1	109.61 (16)	C7—C8—C3	122.10 (18)
C8—N1—H1	125.6 (14)	C2—C9—H9A	109.5
C1—N1—H1	124.7 (14)	C2—C9—H9B	109.5
C2—C1—N1	108.81 (16)	H9A—C9—H9B	109.5
C2—C1—C10	130.42 (16)	C2—C9—H9C	109.5
N1—C1—C10	120.63 (17)	H9A—C9—H9C	109.5
C1—C2—C3	106.81 (16)	H9B—C9—H9C	109.5
C1—C2—C9	128.14 (17)	C15—C10—C11	118.04 (17)
C3—C2—C9	125.05 (17)	C15—C10—C1	121.45 (16)
C4—C3—C8	118.57 (17)	C11—C10—C1	120.48 (16)
C4—C3—C2	133.71 (18)	C12—C11—C10	121.30 (17)
C8—C3—C2	107.71 (17)	C12—C11—H11	119.3
C5—C4—C3	119.44 (18)	C10—C11—H11	119.3
C5—C4—H4	120.3	C13—C12—C11	118.10 (18)
C3—C4—H4	120.3	C13—C12—H12	120.9
C4—C5—C6	120.67 (18)	C11—C12—H12	120.9
C4—C5—H5	119.7	F1—C13—C14	118.92 (17)
C6—C5—H5	119.7	F1—C13—C12	118.33 (17)
C7—C6—C5	121.69 (18)	C14—C13—C12	122.75 (19)
C7—C6—H6	119.2	C13—C14—C15	118.33 (18)
C5—C6—H6	119.2	C13—C14—H14	120.8
C6—C7—C8	117.49 (18)	C15—C14—H14	120.8
C6—C7—H7	121.3	C14—C15—C10	121.43 (18)
C8—C7—H7	121.3	C14—C15—H15	119.3
N1—C8—C7	130.84 (17)	C10—C15—H15	119.3
N1—C8—C3	107.05 (16)		
			
C8—N1—C1—C2	−0.5 (2)	C4—C3—C8—N1	−178.33 (15)
C8—N1—C1—C10	175.64 (15)	C2—C3—C8—N1	0.56 (19)
N1—C1—C2—C3	0.9 (2)	C4—C3—C8—C7	1.7 (3)
C10—C1—C2—C3	−174.81 (17)	C2—C3—C8—C7	−179.37 (16)
N1—C1—C2—C9	−178.84 (17)	C2—C1—C10—C15	−35.0 (3)
C10—C1—C2—C9	5.5 (3)	N1—C1—C10—C15	149.74 (17)
C1—C2—C3—C4	177.78 (19)	C2—C1—C10—C11	143.0 (2)
C9—C2—C3—C4	−2.5 (3)	N1—C1—C10—C11	−32.2 (2)
C1—C2—C3—C8	−0.9 (2)	C15—C10—C11—C12	1.1 (3)
C9—C2—C3—C8	178.83 (16)	C1—C10—C11—C12	−176.98 (16)
C8—C3—C4—C5	−0.6 (3)	C10—C11—C12—C13	0.8 (3)
C2—C3—C4—C5	−179.09 (18)	C11—C12—C13—F1	178.20 (17)
C3—C4—C5—C6	−1.1 (3)	C11—C12—C13—C14	−2.2 (3)
C4—C5—C6—C7	1.6 (3)	F1—C13—C14—C15	−178.86 (16)
C5—C6—C7—C8	−0.5 (3)	C12—C13—C14—C15	1.5 (3)
C1—N1—C8—C7	179.89 (19)	C13—C14—C15—C10	0.5 (3)
C1—N1—C8—C3	0.0 (2)	C11—C10—C15—C14	−1.8 (3)
C6—C7—C8—N1	178.87 (18)	C1—C10—C15—C14	176.27 (17)
C6—C7—C8—C3	−1.2 (3)		

### Hydrogen-bond geometry (Å, °)

**Table:**

Cg is the centroid of the C3–C8 ring.

**Table:**

*D*—H···*A*	*D*—H	H···*A*	*D*···*A*	*D*—H···*A*
N1—H1···Cg^i^	0.94 (2)	2.99 (2)	3.791 (2)	143.(2)

Symmetry codes: (i) *x*, −*y*+1/2, *z*−1/2.

## References

[bb1] Buu-Hoi, N.-H. & Jacquignon, P. (1949). *Recl. Trav. Chim. Pays-Bas*, **68**, 781–788.

[bb2] Konno, T., Chae, J., Ishihara, T. & Yamanaka, H. (2004). *J. Org. Chem.* **69**, 8258–8265. 10.1021/jo048872x 15549796

[bb3] Kraus, G. A. & Guo, H. (2009). *J. Org. Chem.* **74**, 5337–5341. 10.1021/jo900718g 19527008

[bb4] Kumar, M. P. & Liu, R.-S. (2006). *J. Org. Chem.* **71**, 4951–4955. 10.1021/jo0606711 16776526

[bb5] Moriya, T., Takabe, S., Maeda, S., Matsumoto, K., Takashima, K., Mori, T. & Takeyama, S. (1986). *J. Med. Chem.* **29**, 333–341. 10.1021/jm00153a006 3950914

[bb6] Rigaku (2010). *CrystalClear* Rigaku Americas, The Woodlands, Texas, USA, and Rigaku Corporation, Tokyo, Japan.

[bb7] Schmelter, B., Bradaczek, H. & Luger, P. (1973). *Acta Cryst.* B**29**, 971–976.

[bb8] Sheldrick, G. M. (2008). *Acta Cryst.* A**64**, 112–122. 10.1107/S0108767307043930 18156677

